# Histopathological Features of Nevus Sebaceous and Superimposed Skin Tumors

**DOI:** 10.1155/jskc/3828133

**Published:** 2025-05-26

**Authors:** Paridokht Karimian, Naz Zargar Javaheri, Kaveh Gharaei Nejad, Hojat Eftekhari, Elahe Rafiei, Rana Rafiei

**Affiliations:** ^1^Department of Pathology, Medical School, Guilan University of Medical Sciences, Rasht, Iran; ^2^Skin Research Center, Department of Dermatology, Razi Hospital, School of Medicine, Guilan University of Medical Sciences, Rasht, Iran; ^3^Razi Clinical Research Development Unit, Guilan University of Medical Sciences, Rasht, Iran

**Keywords:** benign, malignant, neoplasms, nevus sebaceous, pathology

## Abstract

**Introduction:** Nevus sebaceous (NS) is a rare congenital hamartoma that may cause some concerns for parents. Benign skin tumors can develop in NS, although malignant transformations have occasionally been documented. This study aimed to investigate the histopathological characteristics of NS lesions in relation to sex, age, location, type, and any associated superimposed tumors.

**Methods:** This retrospective cross-sectional study was conducted on the histopathologic reports of 60 patients with NS who were referred to our center between 2010 and 2022. Information, including sex, age, location of the lesion, and histopathological features of the NS lesion, was recorded.

**Results:** The mean age of the patients was 24.52 ± 12.19 years, and 65.0% of the patients (39 cases) were men. Most of the lesions were observed on the scalp and face. Benign and malignant neoplasms were observed in 5% and 10% of patients, respectively. All the cases of malignant tumors (six cases) were identified as basal cell carcinoma, and three cases were benign tumors of syringocystadenoma papilliferum. Demodex infestation and apocrine gland dilatation were observed in 56.7% and 23.3% of patients, respectively. Benign neoplasms were significantly more frequent in women (*p*=0.039). In addition, malignant neoplasms were significantly more prevalent among patients aged ≥ 25 years (*p*=0.001). The frequencies of acanthosis and focal parakeratosis were higher in the scalp, and sebaceous hyperplasia was more common in the face (*p* < 0.05).

**Conclusion:** Our study revealed that malignant tumors were more common than benign lesions in patients with NS. Benign neoplasms were more frequent in women. Malignant neoplasms were detected in patients aged more than 25 years.

## 1. Introduction

Nevus sebaceous (NS), also referred to as organoid nevus, Jadassohn nevus, or pilosyringosebaceous nevus, is classified as a rare, nonhereditary, congenital hamartoma. It arises from hyperplasia of the epithelial, sebaceous, follicular, and apocrine components of the skin [1]. Clinically, NS appears as a solitary, flat, or slightly raised yellowish plaque. The surface of the nevus may be entirely smooth and partially hairless, developing a papillomatous appearance after puberty [2]. The incidence of NS in newborns is estimated to be between 0.1% and 0.3%, with no apparent preference for sex or ethnicity. Despite this, familial cases of NS have been reported [1, 3]. Typically, NS presents as a single lesion at birth, although it may not be recognized until later in life [4]. The lesion grows in proportion to the patient's age but becomes noticeably thicker with a verrucous surface during puberty [5]. The most commonly affected areas include the scalp, followed by the preauricular region, face, and neck. However, various studies have documented its occurrence in other less common locations, such as the mucosa, trunk, and extremities, along the lines of Blaschko [1, 6, 7].

NS has a distinctive clinical presentation, is frequently diagnosed during childhood or adolescence, and results in some concerns in parents. Diagnosis is confirmed by a pathologist after the lesion has been surgically removed [8]. Benign or malignant tumors may develop within NS, typically in adulthood. The most common benign secondary neoplasms are trichoblastoma and syringocystadenoma papilliferum (SCAP), whereas basal cell carcinoma (BCC) is the predominant malignant tumor associated with NS [9, 10]. There is an ongoing debate about the risk of malignant transformation in NS. Earlier research indicated a high incidence of both benign and malignant tumors; however, more recent studies suggest that the occurrence of BCC is rare and that benign tumors develop with a much lower frequency in NS [9, 11]. The pathological features of NS have not been thoroughly examined in the population of northern Iran. The aim of this study was to document the histopathological characteristics of NS, considering sex, age, location, type, and any associated superimposed tumors.

## 2. Methods

This retrospective cross-sectional study was conducted on the histopathologic reports of 60 patients with NS who were referred to our center between 2010 and 2022. The study was approved by the Ethical Committee of Guilan University of Medical Sciences, Rasht, Iran (IR.GUMS.REC.1401.327). In this study, the diagnosis of NS by a dermatologist was based on clinical observations and biopsy. Biopsy samples of patients' skin lesions were evaluated by a dermatopathologist. Patients without a complete pathology report or a definitive diagnosis of NS were not included in the study. The data collected included sex, age, location of the lesion, initial clinical diagnosis, final diagnosis, and histopathological findings such as orthokeratosis, focal parakeratosis, acanthosis, papillomatosis, basal layer pigmentation, sebaceous hyperplasia, terminal hair follicles, secondary neoplasms (malignant or benign), Demodex infestation, epidermal focal hypergranulosis, and dilatation of the apocrine glands.

In this study, the quantitative variables were described with mean ± standard deviations (means ± SDs) and medians (minimum–maximum). Comparisons of the histopathological findings of NS according to sex, age, location, and tumor type were performed via the chi-square test and Fisher's exact test. The data were analyzed via SPSS software Version 21, and the significance level was set as 0.05.

## 3. Results

**Figure 1:**
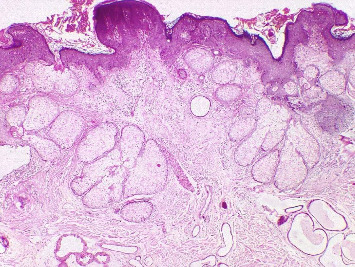
Hyperkeratosis, acanthosis, papillomatosis, and hyperplastic sebaceous glands, which are directly connected to the epidermis and associated with dilated apocrine and eccrine glands in the deep dermis, are present in this sebaceous nevus (hematoxylin and eosin staining × 100).

**Figure 2:**
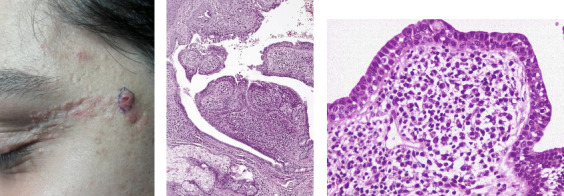
(a) Sebaceous nevus on the lateral canthus of a young patient, a benign tumor of the apocrine gland superimposed on this nevus (a line is drawn around it). (b, c) Syringocystadenoma papilliferum was developed on this nevus. Papillary projections with apocrine decapitation and plasma cell infiltration have been presented in this tumor (hematoxylin and eosin staining × 100, × 400).

The mean age of the patients was 24.52 ± 12.19 years, with a median age of 21.00 years; 39 cases (65.0%) were male, and 50 (83.4%) of the skin lesions were observed on the scalp and face. Forty-four (73.3%) patients were diagnosed with NS on the basis of clinical observations (Table 1). The most frequent histopathological findings were papillomatosis in 51 (85.0%), acanthosis in 49 (81.7%), and orthokeratosis in 45 (75.0%) patients (Figure 1). Spongiosis has not been reported in any patients. Benign neoplasms were found in 3 (5%) patients, and malignant neoplasms were found in 6 (10%) patients. All six cases of malignant tumors were BCC, and three cases were benign tumors of the SCAP (Figure 2). Skin tumors were present in nine patients, with six located on the scalp and three on the face. One patient had both benign and malignant tumors within a single NS lesion. Furthermore, Demodex infestation in 34 (56.7%) patients and apocrine gland dilatation in 14 (23.3%) patients were observed (Table 2).

The frequency of benign neoplasms was greater in women than in men (*p*=0.039). No other significant differences were detected between the sexes (*p* > 0.05). The frequency of malignant neoplasms was significantly higher among patients aged ≥ 25 years (*p*=0.001). Acanthosis and focal parakeratosis were more common on the scalp than on the face (24 [96.0%] vs. 16 [64.0%], *p*=0.005 and 8 [32.0%] vs. 2 [8.0%], *p*=0.034, respectively). Conversely, sebaceous hyperplasia was more common on the face than on the scalp (19 [76.0%] vs. 12 [48.0%], *p*=0.041) (Table 3). The presence of Demodex, epidermal focal hypergranulosis, and dilatation of apocrine glands did not significantly differ between patients with skin neoplasms and those without tumors (*p* > 0.05) (Table 4).

## 4. Discussion

The occurrence, nature, and types of secondary neoplastic changes within NS remain a subject of debate. This study aimed to shed light on the pathological features of NS and investigate potential correlations with demographic and histopathological factors in northern Iran. In our study, the average age of patients at the time of excision was 24.52 ± 12.19 years (range: 8–65), with histopathological evaluations predominantly conducted in individuals under 25 years of age. For comparison, a previous study in Tehran, Iran, reported an average patient age of 21.1 years (range: 2–62) [8]. In addition, the average ages of NS patients in China and the United States of America are 23.5 years (range: 3–60) and 27.4 years (range: 0–95), respectively [12, 13]. In our population, NS was more common in men than in women, a finding that is consistent with some studies [14, 15]. However, other studies have reported a higher prevalence in women [12, 13]. The most frequent locations for NS in our population were the scalp and face, with equal frequency, whereas other studies have identified the scalp as the most common site [1, 13, 16]. The most common histopathological findings, such as papillomatosis, acanthosis, and orthokeratosis, are consistent with previous reports in the literature [15, 17–19]. Notably, acanthosis and focal parakeratosis were more often observed on the scalp, whereas sebaceous hyperplasia was more prevalent on the face.

However, the absence of spongiosis in our study group contrasts with some reports [8, 20]. Unlike the findings of Kamyab-Hesari et al., basal layer hyperpigmentation was less common in our study. Some studies also did not report spongiosis or basal layer hyperpigmentation in NS patients [13, 14], suggesting that these may not be consistent features of NS or may vary in prevalence among populations. Benign and malignant tumors were more frequent in individuals aged 25 years and older, although the difference in benign tumors across age groups was not statistically significant. Another study reported the highest proportion of both benign and malignant neoplasms in the adult age group [13]. Different frequencies have been reported in other studies, with benign tumors ranging from 1.1% to 18% and malignant tumors ranging from 0.8% to 3.5% [13, 14, 21, 22]. In addition, Wilson-Jones and Heyl reported an incidence of benign and malignant tumors as high as 39%, whereas Beer et al. reported a rate of 22.2% [23, 24]. However, the absence of malignant tumors has been reported in some studies [8, 25]. Importantly, samples from NS patients reviewed in the present and past studies were obtained from lesions across different age groups. The development of BCCs or other malignancies within these nevi is likely a time-dependent phenomenon that does not occur unless the lesion has aged sufficiently [8, 16].

The frequency of benign tumors was also greater in women than in men, similar to the findings of Cribier's study (22). However, the difference between malignant tumors and sex was not statistically significant. Idriss and Elston reported that the difference in the proportion of neoplasms between male and female patients was not statistically significant [13]. Consistent with previous studies, SCAP was the most common benign tumor, followed by trichoblastoma, in patients with NS [14, 21, 22, 26]. In contrast, Idriss and Elston reported that trichoblastoma was the most common benign tumor [13]. Consistent with our findings, some studies have also reported that BCC is the most common malignant tumor among patients [9, 13, 27]. However, other carcinomas, such as squamous cell carcinoma and adnexal carcinoma, can also occur [1, 26]. In addition, one of our patients presented with a combination of benign and malignant tumors in a single NS lesion. Similar instances of NS harboring mixed tumors have been reported [13, 26, 28, 29]. The various results concerning the frequency of benign and malignant tumors associated with NS may be attributed to the difficulty in pathologically distinguishing BCC from trichoblastoma. Many trichoblastomas were initially misdiagnosed as BCC but were later identified correctly through a re-evaluation of their histological features (22).

The presence of both benign and malignant neoplasms within NS lesions emphasizes the need for careful monitoring. This finding also suggests the possibility of divergent pathogenic pathways within the same lesion, necessitating comprehensive evaluation and personalized treatment strategies. Consistent with other studies, Demodex infestation and apocrine gland dilatation were noted in NS patients [19, 30]. However, we found no significant differences in these conditions between individuals with and without skin neoplasms. Sun et al. suggested that BCC cases presented the highest rate of Demodex infestation among the four types of facial neoplasms [31].

Also, the deregulation of patched-hedgehog signaling pathway as a tumor suppressor gene has been implicated in the development of malignant transformation such as BCC in NS. Tojo, et al. found that trichoblastomas, which were superimposed on NS, did not show loss of heterozygosity at the patched gene. Therefore, trichoblastoma as a benign tumor has a different molecular pathogenesis from BCC. More frequent presentation of BCC in our study may be explained by genetic variations in different populations [32, 33].

This finding that malignant tumors were more common than benign lesions in patients with NS may be in contrast with some previous literature. This could be due to the small sample of patients available or to the fact that, in the clinical suspicion of a neoplastic degeneration towards malignancy, there is a greater tendency to perform excision.

Considering the increased risk of malignant tumors with aging, regular follow-up of NS with noninvasive methods such as dermoscopy has been recommended for children [1].

The inclusion of various clinicopathological aspects of NS provides a comprehensive analysis of NS characteristics. However, it is important to recognize the limitations of this study. The study was conducted in a geographically limited area (northern Iran), which may affect the generalizability of the findings. The retrospective design of the study introduces a potential selection bias, as only patients who visited the specific center were included. In addition, reliance on existing medical biopsy reports may introduce inconsistencies and potential errors in data interpretation.

Prospective studies with longitudinal follow-up would be beneficial for understanding the natural history and progression of NS. Multicenter studies with diverse populations would help confirm the generalizability of the results.

## 5. Conclusion

Our study revealed that the majority of secondary neoplasms associated with NS were malignant. Malignant neoplasms were observed in individuals aged 25 years and older, and benign neoplasms were more common in women.

The finding of a greater incidence of malignant neoplasms in patients aged over 25 years highlights the importance of monitoring these lesions, particularly in older individuals. It would be beneficial to propose a more standardized approach to follow-up care and screening for NS lesions, including the use of dermoscopy and other noninvasive methods such as reflectance confocal microscopy and line-field confocal optical coherence tomography especially for at-risk patients.

## Figures and Tables

**Table 1:** Demographic characteristics and clinical diagnosis of NS among patients (*N* = 60).

Variables		*N* (%)
Age (years)	< 25	38 (63.3)
	≥ 25	22 (36.7)

Gender	Male	39 (65.0)
	Female	21 (35)

Location of the lesion	Scalp	25 (41.7)
	Face	25 (41.7)
	Trunk	1 (1.7)
	Unknown	9 (15.0)

Clinical diagnosis of NS	Yes	44 (73.3)
	No	6 (10.0)
	Without clinical data	10 (16.7)

*Note: N*: number.

Abbreviation: NS, nevus sebaceous.

**Table 2:** Histopathological findings of NS lesions among patients (*N* = 60).

Histopathological findings		*N* (%)
Orthokeratosis	Yes	45 (75.0)
	No	15 (25.0)

Focal parakeratosis	Yes	12 (20.0)
	No	48 (80.0)

Focal hypergranulosis	Yes	14 (23.3)
	No	46 (76.7)

Acanthosis	Yes	49 (81.7)
	No	11 (18.3)

Papillomatosis	Yes	51 (85.0)
	No	9 (15.0)

Basal layer pigmentation	Yes	10 (16.7)
	No	50 (83.3)

Sebaceous hyperplasia	Yes	37 (61.7)
	No	23 (38.3)

Terminal hair follicles	Yes	27 (45.0)
	No	33 (55.0)

Benign neoplasm	Yes	3 (5.0)
	No	57 (95.0)

Malignant neoplasm	Yes	6 (10.0)
	No	54 (90.0)

Demodex infestation	Yes	34 (56.7)
	No	26 (43.3)

Apocrine glands dilatation	Yes	14 (23.3)
	No	46 (76.7)

*Note: N*: number.

**Table 3:** Histopathological findings of NS lesions according to sex, age, and location.

Histopathological findings		Gender		*p* value	Age		*p* value	Location		*p* value
		Female (*n* = 21)	Male (*n* = 39)		< 25 (*n* = 38)	≥ 25 (*n* = 22)		Scalp (*n* = 25)	Face (*n* = 25)	
Orthokeratosis	Yes	16 (76.2)	29 (74.4)	0.876^∗^	28 (73.7)	17 (77.3)	0.757^∗^	19 (76.0)	18 (72.0)	0.747^∗^
	No	5 (23.8)	10 (25.6)		10 (26.3)	5 (22.7)		6 (24.0)	7 (28.0)	

Focal parakeratosis	Yes	3 (14.3)	9 (23.1)	0.513^∗∗^	6 (15.8)	6 (27.3)	0.327^∗∗^	8 (32.0)	2 (8.0)	0.034^∗^
	No	18 (85.7)	30 (76.9)		32 (84.2)	16 (72.7)		17 (68.0)	23 (92.0)	

Focal hypergranulosis	Yes	6 (28.6)	8 (20.5)	0.532^∗∗^	8 (21.1)	6 (27.3)	0.583^∗^	6 (24.0)	6 (24.0)	> 0.999^∗^
	No	15 (71.4)	31 (79.5)		30 (78.9)	16 (72.7)		19 (76.0)	19 (76.0)	

Acanthosis	Yes	16 (76.2)	33 (84.6)	0.493^∗∗^	31 (81.6)	18 (81.8)	> 0.999^∗∗^	24 (96.0)	16 (64.0)	0.005^∗^
	No	5 (23.8)	6 (15.4)		7 (18.4)	4 (18.2)		1 (4.0)	9 (36.0)	

Papillomatosis	Yes	17 (81.0)	34 (87.2)	0.706^∗∗^	32 (84.2)	19 (86.4)	> 0.999^∗∗^	23 (92.0)	19 (76.0)	0.247^∗∗^
	No	4 (19.0)	5 (12.8)		6 (15.8)	3 (13.6)		2 (8.0)	6 (24.0)	

Basal layer pigmentation	Yes	5 (23.8)	5 (12.8)	0.298^∗∗^	6 (15.8)	4 (18.2)	> 0.999^∗∗^	6 (24.0)	3 (12.0)	0.463^∗∗^
	No	16 (76.2)	34 (87.2)		32 (84.2)	18 (81.8)		19 (76.0)	22 (88.0)	

Sebaceous hyperplasia	Yes	12 (57.1)	25 (64.1)	0.597^∗^	22 (57.9)	15 (68.2)	0.430^∗^	12 (48.0)	19 (76.0)	0.041^∗^
	No	9 (42.9)	14 (35.9)		16 (42.1)	7 (31.8)		13 (52.0)	6 (24.0)	

Terminal hair follicles	Yes	10 (47.6)	17 (43.6)	0.765^∗^	17 (44.7)	10 (45.5)	0.957^∗^	15 (60.0)	9 (36.0)	0.089^∗^
	No	11 (52.4)	22 (56.4)		21 (55.3)	12 (54.5)		10 (40.0)	16 (64.0)	

Benign neoplasm	Yes	3 (14.3)	0 (0)	0.039^∗∗^	1 (2.6)	2 (9.1)	0.548^∗∗^	1 (4.0)	2 (8.0)	> 0.999^∗∗^
	No	18 (85.7)	39 (100.0)		37 (97.4)	20 (90.9)		24 (96.0)	23 (92.0)	

Malignant neoplasm	Yes	4 (19.0)	2 (5.1)	0.171^∗∗^	0 (0)	6 (27.3)	0.001^∗∗^	5 (20.0)	1 (4.0)	0.189^∗∗^
	No	17 (81.0)	37 (94.9)		38 (100.0)	16 (72.7)		20 (80.0)	24 (96.0)	

Demodex infestation	Yes	10 (47.6)	24 (61.5)	0.299^∗^	21 (55.3)	13 (59.1)	0.773^∗^	14 (56.0)	16 (64.0)	0.564^∗^
	No	11 (52.4)	15 (38.5)		17 (44.7)	9 (40.9)		11 (44.0)	9 (36.0)	

Apocrine glands dilatation	Yes	5 (23.8)	9 (23.1)	> 0.999^∗∗^	11 (28.9)	3 (13.6)	0.177^∗^	7 (28.0)	5 (20.0)	0.508^∗^
	No	16 (76.2)	90 (76.9)		27 (71.1)	19 (86.4)		18 (72.0)	20 (80.0)	

*Note: N*: number.

^∗^ *q* square test; a *p* value of less than 0.05 was considered statistically significant.

^∗∗^Fisher's exact test.

**Table 4:** The frequency of some histopathological findings of NS lesions according to the presence of skin tumors.

Histopathological findings		Skin tumors		*p* value^∗^
		Yes (*n* = 8)	No (*n* = 52)	
Demodex infestation	Yes	4 (50.0)	30 (57.7)	0.717
	No	4 (50.0)	22 (42.3)	

Focal hypergranulosis	Yes	3 (37.5)	11 (21.2)	0.374
	No	5 (62.5)	41 (78.8)	

Apocrine glands dilatation	Yes	3 (37.5)	11 (21.2)	0.374
	No	5 (62.5)	41 (78.8)	

*Note: N*: number.

^∗^Fisher's exact test; a *p* value of less than 0.05 was considered statistically significant.

## Data Availability

The datasets used and/or analyzed during the present study are available from the corresponding author upon reasonable request.
